# Clinical Spectrum of Hyponatremia in Patients with Stroke

**DOI:** 10.7759/cureus.5310

**Published:** 2019-08-02

**Authors:** Moiz Ehtesham, Mawa Mohmand, Kuldeep Raj, Tooba Hussain, FNU Kavita, Besham Kumar

**Affiliations:** 1 Internal Medicine, Dow International Medical College, Dow University of Health Sciences, Karachi, PAK; 2 Internal Medicine, Khyber Medical College, Peshawar, PAK; 3 Internal Medicine, Jinnah Sindh Medical University, Karachi, PAK; 4 Medicine, Dow University of Health Sciences, Karachi, PAK; 5 Medicine, Liaquat University of Medical and Health Sciences, Jamshoro, PAK; 6 Internal Medicine, Jinnah Postgraduate Medical Center, Karachi, PAK

**Keywords:** stroke, hyponatremia, ischemic stroke, hemorrhagic stroke, severity, frequency

## Abstract

Introduction

Hyponatremia is a common electrolyte imbalance, which is readily observed in patients with ischemic as well as hemorrhagic stroke. It is mostly hypoosmolal and may be due to syndrome of inappropriate anti-diuretic hormone (SIADH) or cerebral salt wasting syndrome (CSWS). The aim of this study was to evaluate the clinical spectrum of hyponatremia in patients of both hemorrhagic and ischemic strokes.

Methods

In this prospective observational study, all patients admitted with stroke were screened for serum sodium levels right after hospital admission. Patients with serum sodium levels <135 mEq/L were included. Their demographic characteristics, type of stroke, etiology of hyponatremia, and site of hemorrhage/vascular territory ischemia was included.

Results

Hyponatremia was diagnosed in 34.2% of patients. Their mean serum sodium level was 130.4 ± 3.5 (mEq/L). Ischemic stroke was more common in the hyponatremia group (67.7%), and SIADH was a more common cause of hyponatremia (71.1%). In hyponatremic patients with hemorrhagic stroke, right putamen hemorrhage was seen in 50% of patients with SIADH, and right thalamus was seen in 73.3% patients with CSWS. In hyponatremic patients with ischemic stroke, left middle cerebral artery ischemia was seen in 47% patients with SIADH and right middle cerebral artery ischemia was seen in 55% patients with CSWS.

Conclusion

In patients with hyponatremia secondary to stroke, ischemic stroke is a common entity. SIADH remains a more frequently witnessed underlying pathology in hyponatremic stroke patients.

## Introduction

Hyponatremia is a common electrolyte imbalance which is readily observed in patients of neurological disorders such as stroke. Hyponatremia is defined as serum sodium levels ≤135 mmol/L [1]. In stroke, hyponatremia is mostly hypoosmolal and may be caused by either syndrome of inappropriate anti-diuretic hormone (SIADH) or cerebral salt wasting syndrome (CSWS). SIADH is more common than CSWS [2]. It may also be due to dietary restriction of sodium as a measure to control hypertension, use of anti-hypertensive medicine such as diuretics, and secondary infections. It results in altered sensorium in these patients and may also predispose the patients to seizures.

In SIADH, there is an unchecked secretion of anti-diuretic hormone (ADH), from the posterior pituitary gland in response to stimulation from the hypothalamus. It causes body fluid hypotonicity and increased blood volume [2]. In CSWS, large quantities of sodium are lost in the urine [3].

Incidence of hyponatremia in stroke has been reported to be 11% to 35% in the literature [2,4-5]. The rate of mortality in hyponatremic stroke patients has been reported to be as high as 60% [4]. In Pakistani literature, the frequency of hyponatremia has been reported to be as high as 35% to 45% in stroke patients; however, the mortality rate has been lower (16%) as compared to other literature [6-7]. Mahesar et al. stated that only patients were ischemic stroke were included, and Shah et al. included only patients with hemorrhagic stroke [6-7]. In this study, we aimed to establish the frequency and severity of hyponatremia in patients of both hemorrhagic and ischemic strokes of all etiologies.

## Materials and methods

A prospective observational study was conducted in the Department of Neuromedicine of a tertiary care public hospital in Karachi. The study duration was 1st July to 31st December 2018. This study was approved by the institutional review board. Informed consent was taken from all patients.

During the study period, 354 patients, of both genders and age 18 years and above, were admitted with a clinical and neuro-radiological diagnosis of stroke. Strokes were classified as ischemic or hemorrhagic based on the findings of neuro-imaging by experienced neurologists. Hyponatremia was defined as serum sodium concentration <135 mmol/L just after admission prior to administration of any medical therapy. The reason for this was that late-onset hyponatremia may be iatrogenic and not purely a consequence of the stroke itself. The underlying etiology was classified as SIADH or CSWS based on the classification described in Saleem et al. [2].

In patients with hemorrhagic stroke, the site of hemorrhage was identified on magnetic resonance imaging (MRI) or magnetic resonance angiography (MRA). The site of ischemia was determined on MRA. Where MRI or MRA was not possible or contraindicated, non-contrast computed tomography (NCCT) was done. SPSS for Windows version 22.0 (IBM Corp., Armonk, NY, US) was used to process the data.

## Results

There were 226 (63.8%) male and 128 (36.2%) female patients. Their mean age was 28 ± 11 years (range: 24-68 years). There were 112 (31.6%) patients of age 18-45 years. All patients were admitted from the emergency room. Hyponatremia was diagnosed in 121 (34.2%) patients. The mean serum sodium level of these hyponatremic patients was 130.4 ± 3.5 (mEq/L). 

The characteristics of patients with hyponatremia are shown in Table 1. More patients were males and of older age group. Ischemic stroke was more common among hyponatremic patients and SIADH was a more common cause of hyponatremia, as seen in Table 1.

**Table 1:** Characteristics of stroke patients with hyponatremia (N = 121) SIADH, syndrome of inappropriate secretion of antidiuretic hormone; CSWS, cerebral salt wasting syndrome

Patient Characteristics	Frequency (%)
Gender	
Male	73 (60.3%)
Female	48 (39.7%)
Age in years	
18-45 years	42 (34.7%)
>45 years	79 (65.3%)
Known comorbidity status	
Neurological disease	48 (39.6%)
Hypertension	28 (23.1%)
Ischemic heart disease	17 (14.1%)
Diabetes mellitus	9 (7.4%)
Type of stroke	
Hemorrhagic stroke	39 (32.2%)
Ischemic stroke	82 (67.7%)
Cause of hyponatremia	
SIADH	86 (71.1%)
CSWS	35 (28.9%)

Among ischemic stroke patients with hyponatremia, SAIDH was established in 62 (75.6%) patients and CSWS in 20 (24.4%) patients. Comparatively, in hemorrhagic stroke patients with hyponatremia, SAIDH was established in 24 (61.5%) patients and CSWS in 15 (38.5%) patients. The mean serum sodium level in the ischemic group of hyponatremia was 128.2 ± 1.8 (mEq/L) and that of the hemorrhagic group was 124.7 ± 2.5 (mEq/L).

The site of hemorrhage was then determined in patients with hemorrhagic stroke, as shown in Table 2. Right putamen was the most common site of hemorrhage in patients with SIADH and right thalamus was the most common in patients with CSWS. None of the patients in the SIADH group had pontine hemorrhage and only one in CSWS group had it (Table 2).

**Table 2:** Site of hemorrhage in hyponatremic patients with hemorrhagic stroke (N = 39) SIADH, syndrome of inappropriate secretion of antidiuretic hormone; CSWS; cerebral salt wasting syndrome

Sites of hemorrhagic stroke	Hyponatremia due to SIADH (n = 24)	Hyponatremia due to CSWS (n = 15)
Right putamen hemorrhage	12 (50%)	7 (46.7%)
Left putamen hemorrhage	9 (37.5%)	9 (60%)
Right thalamic hemorrhage	3 (12.5%)	11 (73.3%)
Left thalamic hemorrhage	8 (33.3%)	6 (40%)
Right cerebellar hemorrhage	2 (8.3%)	3 (20%)
Left cerebellar hemorrhage	1 (4.2%)	---
Pontine hemorrhage	---	1 (6.7%)

Of all the patients of ischemic stroke with hyponatremia (n = 82), left middle cerebral artery (MCA) ischemia was the most common (n = 35/82; 42.6%), followed by right MCA (n = 33/82; 40.2%), and ischemia of anterior circulation was the least frequent (n = 14/82; 17.1%). The distribution of ischemic territory according to the type of hyponatremia is shown in Table 3.

**Table 3:** Ischemic vascular territory in hyponatremic patients with ischemic stroke (N = 82) SIADH, syndrome of inappropriate secretion of antidiuretic hormone; CSWS, cerebral salt wasting syndrome

Vascular Territory involved	Hyponatremia due to SIADH (n = 62)	Hyponatremia due to CSWS (n = 20)
Right Middle Cerebral Artery	22 (35.4%)	11 (55%)
Left Middle Cerebral Artery	29 (46.7%)	6 (30%)
Anterior Circulation	11 (17.7%)	3 (15%)

Out of 121 hyponatremic patients, 81 (66.9%) had mild hyponatremia (serum sodium: 130-134 mEq/L) and 62 (76.5%) of them did not show any changes in mental and physical functioning. The remaining 29 (35.8%) patients complained of generalized lethargy, malaise, excessive sleepiness, and nausea. Among the 31 (25.6%) patients of moderate hyponatremia (serum sodium: 125-130 mEq/L), 22 (70.9%) developed altered mental state, six (19.3%) patients were lethargic with deep sleep (but not in coma), and three (9.7%) patients developed pulmonary edema. Among the nine (7.4%) patients with severe hyponatremia (serum sodium: <125 mEq/L), all patients developed an altered mental state, three (33.3%) of them had an episode of generalized seizure, and two (22.2%) patients developed cerebral edema.

## Discussion

In this large-scale, single-center prospective study, the frequency of hyponatremia in stroke has been reported to be comparable to other data from the same region [6-7]. SIADH was reported as a more common entity. Ischemic strokes were more common than hemorrhagic ones. As with Shah et al., this study did not follow the patients for their hospital outcome and did not aim to establish any relationship between hyponatremia and mortality, which is a major limitation [7]. This study was time-bound with a limited number of patients.

Hyponatremia remains the most common electrolyte imbalance encountered in hospitalized patients. It is more frequently seen in patients with intracranial, both medical and surgical, disorders and severely ill patients admitted in the intensive care units. It may be due to the underlying disease; however, it may also be iatrogenic [8-9]. In most patients with neurologic disorders, hyponatremia is dilutional along with hypoosmolal serum, which induces cerebral edema. In hypertensive patients presenting with stroke, hyponatremia may also be due to pre-stroke diuretic therapy; however, no relationship has been established [9-10]. In an Iraqi study with 17% frequency of hyponatremia in stroke patients, all patients of hyponatremia suffered hemorrhagic stroke. Their clinical presentation included disturbed consciousness, headache, visual disturbances, and convulsions [11].

SIADH has been reported as a more common etiology in hyponatremia as compared to CSWS in the literature. In an Iraqi study with hyponatremic stroke patients, 82% had SIADH and 18% had CSWS [11]. In another study with a frequency of 35% hyponatremia in patients with stroke, 67.5% had SIADH and 32.5% had CSWS. In hemorrhagic stroke group, 67% had SIADH and 33% had CSWS. In the ischemic stroke group, 68.5% had SIADH and 31.5% had CSWS [2]. The results were comparable to our study. Pradhan et al. showed that mean sodium in the hemorrhagic group was lower than that in the ischemic group [12]. 

In this study, right putamen hemorrhage was more common in patients with SIADH and right thalamus hemorrhage was more common in patients with CSWS. None of the patients in the SIADH group had pontine hemorrhage. In hyponatremic ischemic stroke patients left MCA ischemia was the most common overall as well as in SIADH group. In the CSWS group, right MCA ischemia was more common. Saleem et al. also categorized their hyponatremic patients according to their site of hemorrhage and territory of vascular ischemia. Right MCA ischemia was the most common in CSWS group and left MCA was most commonly involved in the SIADH group. Left putamen hemorrhage was the most common in SIADH group and in CSWS group right putamen and right thalamus hemorrhage was equally seen. Overall, pontine hemorrhage was more common in their study as compared to ours [2]. Shah et al., in their patients with hemorrhagic stroke, reported right putamen hemorrhage to be more common in the SIADH group and left putamen in their CSWS group. The overall incidence of hyponatremia in their study was high - 45% - with 58.5% cases of SIADH and 41.5% of CSWS [7]. Mahesar et al. stated that the frequency of hyponatremia in patients with ischemic stroke was 38.6%. Out of these cases, 64.7% had mild hyponatremia, 25.5% had moderate, and 9.8% had severe hyponatremia [6].

Neurological disorders, including stroke, are frequently complicated by electrolyte imbalance. Serum sodium derangement remains the most commonly witnessed electrolyte imbalance in these patients. Hyponatremia is of prime clinical importance as its clinical presentation may mask the signs of already sustained neurological trauma in these patients. Early identification of rapidly declining serum sodium levels helps in initiating immediate measures in these patients.

## Conclusions

Hyponatremia is a readily observed entity in patients with neurological disorders. In patients with hyponatremia secondary to stroke, ischemic stroke is a common entity. SIADH remains a more frequently witnessed underlying pathology in hyponatremic stroke patients. Severe hyponatremia may further deteriorate the neurological status of these patients. Early identification of rapidly declining serum sodium levels may help in immediate management.
